# Protein Discovery: Combined Transcriptomic and Proteomic Analyses of Venom from the Endoparasitoid *Cotesia chilonis* (Hymenoptera: Braconidae)

**DOI:** 10.3390/toxins9040135

**Published:** 2017-04-12

**Authors:** Zi-Wen Teng, Shi-Jiao Xiong, Gang Xu, Shi-Yu Gan, Xuan Chen, David Stanley, Zhi-Chao Yan, Gong-Yin Ye, Qi Fang

**Affiliations:** 1 State Key Laboratory of Rice Biology & Key Laboratory of Agricultural Entomology of Ministry of Agriculture, Institute of Insect Sciences, Zhejiang University, Hangzhou 310058, China; tzwbat@126.com (Z.-W.T.); xiongshijiao@zju.edu.cn (S.-J.X.); xugang0503@126.com (G.X.); 3120100103@zju.edu.cn (S.-Y.G.); 3120100105@zju.edu.cn (X.C.); yan_zc@126.com (Z.-C.Y.); chu@zju.edu.cn (G.-Y.Y.); 2 USDA/Agricultural Research Service Biological Control of Insects Research Laboratory, Columbia, MO 65203, USA; stanleyd@missouri.edu

**Keywords:** parasitoid wasps, hosts, venom proteins, venom gland, transcriptomics, proteomics

## Abstract

Many species of endoparasitoid wasps provide biological control services in agroecosystems. Although there is a great deal of information on the ecology and physiology of host/parasitoid interactions, relatively little is known about the protein composition of venom and how specific venom proteins influence physiological systems within host insects. This is a crucial gap in our knowledge because venom proteins act in modulating host physiology in ways that favor parasitoid development. Here, we identified 37 possible venom proteins from the polydnavirus-carrying endoparasitoid *Cotesia chilonis* by combining transcriptomic and proteomic analyses. The most abundant proteins were hydrolases, such as proteases, peptidases, esterases, glycosyl hydrolase, and endonucleases. Some components are classical parasitoid venom proteins with known functions, including extracellular superoxide dismutase 3, serine protease inhibitor and calreticulin. The venom contains novel proteins, not recorded from any other parasitoid species, including tolloid-like proteins, chitooligosaccharidolytic β-*N*-acetylglucosaminidase, FK506-binding protein 14, corticotropin-releasing factor-binding protein and vascular endothelial growth factor receptor 2. These new data generate hypotheses and provide a platform for functional analysis of venom components.

## 1. Introduction

Most hymenopteran parasitoids are very small insects that produce minute quantities of venom, which helps explain why research in this area is limited to about 70 of the approximately 300,000 venom-producing species, mainly stinging wasps, bees, and ants [1,2]. Beyond understanding the mechanisms of host/parasitoid relationships, research into parasitoid venom has the potential to uncover a wealth of biomolecules of value in agriculture and pharmacology [1,3,4].

Parasitoid venoms are highly diverse among species [2]. Generally, venoms from ectoparasitoids result in short- to long-term paralysis/lethargy in hosts [5,6]. Compounds identified from ectoparasitoid venom include gamma-aminobutyric acid, β alanine and taurine from *Ampulex compressa* (Hymenoptera, Ampulicidae) [5] and philanthotoxins from *Philanthus triangulum* (Hymenoptera: Crabronidae) [6]. Ectoparasitoid venoms alter host biology, including immunosuppression, developmental arrest, apoptosis, stress response, and regulating metabolism [7,8]. Endoparasitoid venoms also induce transient paralysis, as seen in two ichneumonids, *Pimpla hypochondriaca* (Hymenoptera: Ichneumonidae) [9], and *Pimpla turionellae* (Hymenoptera: Ichneumonidae) [10], as well as in four braconid wasps, including *Asobara tabida* (Hymenoptera: Braconidae) [11], two *Binodoxys* species (Hymenoptera: Braconidae) [12], and *Chelonus inanitus* (Hymenoptera: Braconidae) [13]. One paralytic factor, pimplin, has been purified from *Pimpla hypochondriaca* venom [9]. Transient host paralysis may be an adaptation to reduce self-superparasitism [12]. Endoparasitoid venom can lead to permanent paralysis, for example, *Asobara japonica* (Hymenoptera: Braconidae) venom induces permanent paralysis that leads to the death of *Drosophila melanogaster* (Diptera: Drosophilidae) larvae. The paralysis can be experimentally reversed by injecting unfractionated ovarian extracts from female wasps, although this biological significance of the observation is unclear [14]. Endoparasitoid venom proteins can also influence host cellular immunity, thought to protect their progeny from host immune responses [1,3,15]. Known immunosuppressive venom components include P4 (containing a RhoGAP domain) from *Leptopilina boulardi* (Hymenoptera: Eucoilidae) [16,17], calreticulin (CRT) from *Cotesia rubecula* (Hymenoptera: Braconidae) [18], VPr1, VPr3 from *Pimpla hypochondriaca* [19], Vn.11 from *Pteromalus puparum* (Hymenoptera: Pteromalidae) [20], and sarco/endoplasmic reticulum calcium ATPase (SERCA) from *Ganaspis* sp.1 (Hymenoptera: Figitidae) [21]. Other venom proteins and peptides, including Vn4.6 [22] and Vn50 [23] from *Cotesia rubecula*, LbSPNy [24] and extracellular Cu, Zn-superoxide dismutase [25] from *Leptopilina* species inhibit host melanization. Endoparasitoid venoms also influence host development. *Glyptapanteles liparidis* (Hymenoptera: Ichneumonidae) venom results in prolonged larval development and higher pupal mortality of *Lymantria dispar* (Lepidoptera: Lymantriidae) [26], and *Pteromalus puparum* venom is an endocrine disrupter of *Pieris rapae* (Lepidoptera: Pieridae) [27]. A venom γ-glutamyl transpeptidase of the braconid *Aphidius ervi* (Hymenoptera: Braconidae) castrates the host *Acyrthosiphon pisum* (Homoptera: Aphididae) by inducing apoptosis in host ovarioles. This may re-direct nutrient flow from ovaries to the hemolymph, possibly advantaging developing parasitoids [1,28,29]. On the other hand, in some polydnavirus (PDV)-carrying endoparasitoids, venoms synergize the effect of PDVs in immunosuppression [30,31] and developmental arrest [13,32], probably by promoting persistence and expression of PDVs [31,33].

Combined transcriptomic and proteomic analyses were first used to characterize *Chelonus inanitus* venom proteins, which discriminated between proteins in the venom gland cells and those in the venom [4,34]. This approach has led to insights into venoms from *Chelonus inanitus*, *Hyposoter didymator* (Hymenoptera: Ichneumonidae), *Leptopilina boulardi*, *Leptopilina heterotoma*, *Microplitis demolitor* (Hymenoptera: Braconidae), *Aphidius ervi*, and *Toxoneuron nigriceps* (Hymenoptera: Braconidae) [2,4,35,36,37,38]. Nonetheless, in view of the very large number of parasitoid species, very little is known about the diversity, compositions, and functions of their many venoms. Analysis of venom proteins from a phylogenetically-broad range of parasitoid species will lead to the discovery of new proteins, new chemistries, and valuable information on host/parasitoid relationships.

*Cotesia chilonis* (Hymenoptera: Braconidae) is an obligate larval parasitoid that effectively regulates *Chilo suppressalis* populations (Lepidoptera: Crambidae), an important rice pest in Asia, Northern Africa, and Southern Europe [39]. It is widely distributed in China, Japan, Indonesia, and North Korea where the natural parasitism rates range from 10% to 30% and may be as high as 90%, for example, in Jiangsu province, China [40,41]. The *Cotesia chilonis*/*Chilo suppressalis* relationship is our model host/parasitoid system, from which we reported that *Cotesia chilonis* parasitization influences the expression of host fat body and hemocyte genes [39]. Transcriptome analysis of adult parasitoids revealed chemoreception-related and bracovirus-related genes may be involved in successful parasitism [41]. We found that *Cotesia chilonis* venom inhibits host humoral immunity and it synergizes the immunosuppressive effects of calyx fluid [42]. Here we report the identification of venom proteins consistent with the biology of known parasitoid venoms and several novel proteins, not previously reported from parasitoid wasps. These findings generate new hypotheses and provide a platform for functional analysis of parasitoid venom components.

## 2. Results and Discussion

### 2.1. Transcriptome Analysis of *Cotesia chilonis* Venom Glands

Transcriptome sequence data were generated from two cDNA libraries, a venom gland library (VG) and an adult female carcass (without venom gland) library (FAC). We acquired 67,097,344 bp raw reads from the VG and 63,686,328 bp raw reads from the FAC. After removing reads containing adapter sequences, poly-N sequences and low quality reads, 65,515,114 (VG) and 62,221,866 bp (FAC) clean reads remained (Table 1). The clean reads were assembled using Trinity software [43]. Total clean base pairs yielded 115,957 transcripts with an N50 length of 2393 bp and a mean length of 1254 bp and 71,617 unigenes with an N50 length of 1309 bp and a mean length of 759 bp (Table 1). The *q*-value < 0.005 and |log2(fold change)| > 1 was set as the threshold for significant differential expression. By these criteria, 479 VG unigenes were upregulated compared to FAC unigenes, defined here as upregulated VG unigenes (UVG) (Supplementary Table S1).

We annotated 23,613 unigenes (32.97%) in GO. At level 2, UVG were classified into nine molecular functional categories (Table 2), among which “structural molecule activity” (GO:0003735) and “structural constituent of ribosome” (GO:0003735) categories were highly represented (42 and 37 unigenes respectively). This may relate to gene transcription and translation. Table 2 shows proteins functionally annotated as “peptidase activity” (GO:0008233), “metallopeptidase activity” (GO:0008237), “hydrolase activity, hydrolyzing *O*-glycosyl compounds” (GO:0004553), “hydrolase activity, acting on glycosyl bonds” (GO:0016798), “phospholipase activity” (GO:0004620), and “phospholipase C activity” (GO:0004629). Venom proteins containing conserved enzymatic domains constitute a large proportion of venom components, although their biological roles are not clear in most cases [4,34].

### 2.2. Identification of Secreted Proteins

**Figure 1:**
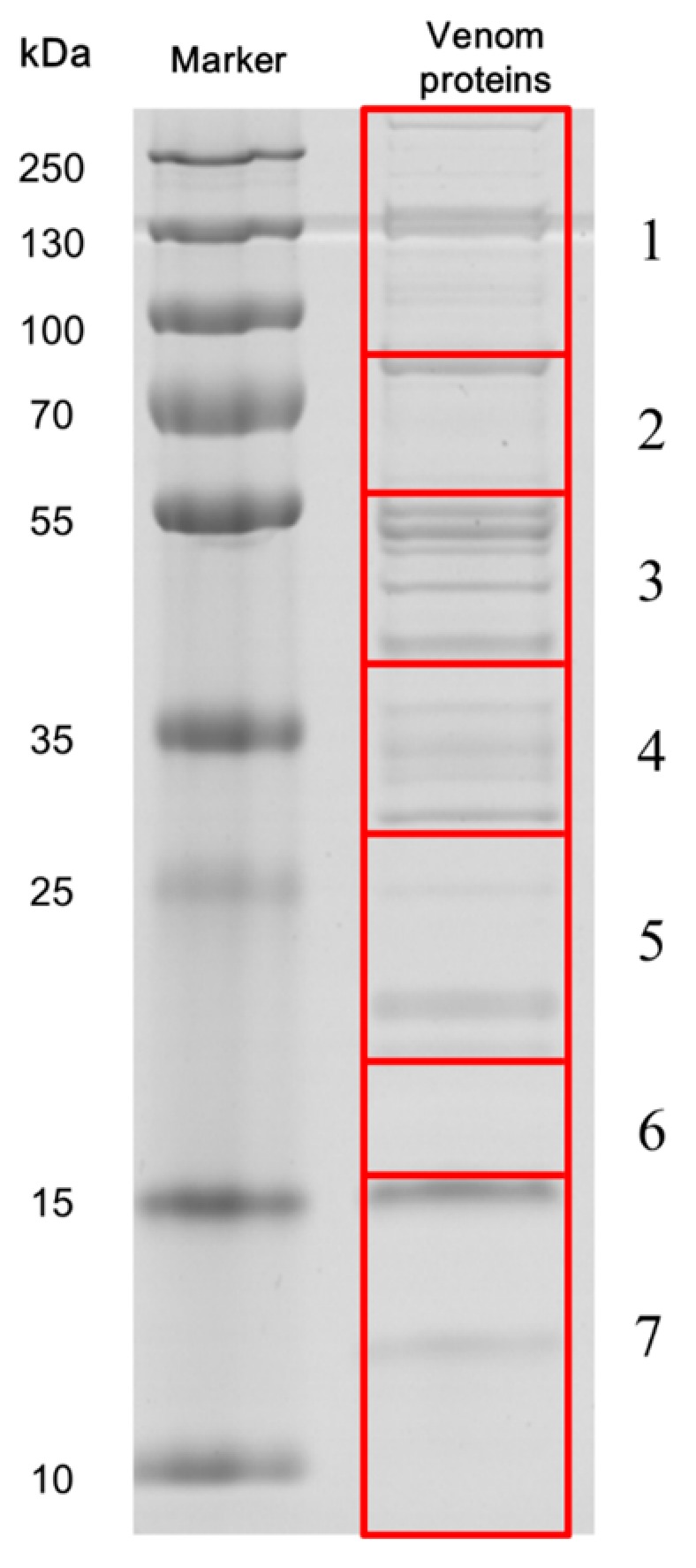
SDS-PAGE analysis of *Cotesia chilonis* venom proteins. Proteins were separated on a 12% SDS-PAGE gel and stained with Coomassie Brilliant Blue R-250. The left lane shows molecular weight markers and the right lane shows the venom proteins. The gel was cut into seven large sections, indicated by the red boxes. Tryptic peptides extracted from each section were excised and analysed on LC-MS/MS.

**Figure 2:**
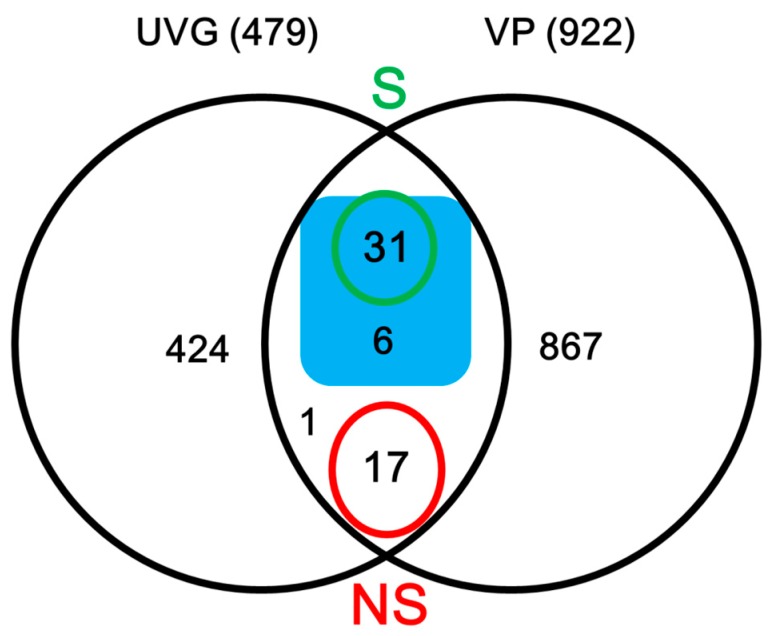
Venn diagram illustrating a likely set of venom proteins. The diagram shows the intersection of UVG unigenes and those matched to the proteomic databases (VP). The numbers in the green and red ellipses represent the numbers of unigenes with complete sequences encoding secreted (green; S) or not secreted proteins (red; NS). The numbers in blue rectangle correspond to the numbers of proteins considered “possible venom proteins” [1,45].

**Figure 3:**
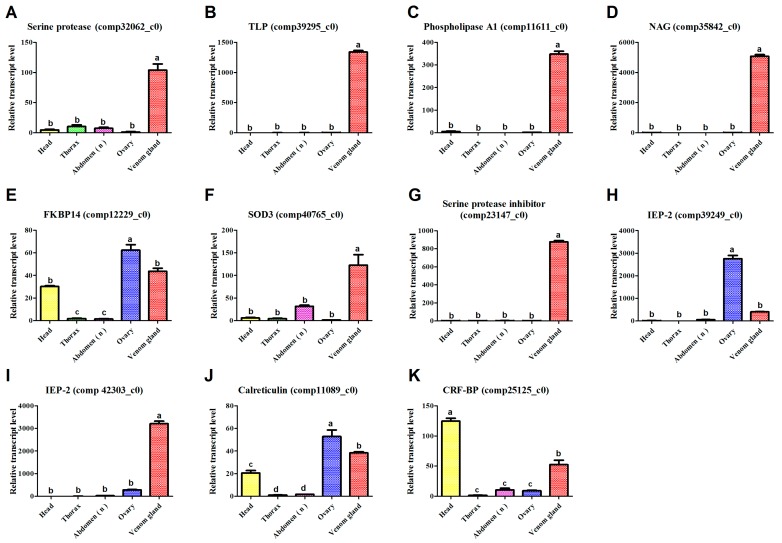
qPCR results showing the mRNA abundance levels of selected unigenes in indicated female tissues. (**A**) Cc-Ven1, Serine protease; (**B**) Cc-Ven4, Tolloid-like protein; (**C**) Cc-Ven10, Phospholipase A1; (**D**) Cc-Ven12, Chitooligosaccharidolytic β-*N*-acetylglucosaminidase; (**E**) Cc-Ven14, FK506-binding protein 14; (**F**) Cc-Ven17, Extracellular superoxide dismutase 3; (**G**) Cc-Ven19, Serine protease inhibitor (Serpin); (**H**) Cc-Ven20, Immunoevasive protein-2; (**I**) Cc-Ven21, Immunoevasive protein-2; (**J**) Cc-Ven22, Calreticulin; (**K**) Cc-Ven25, Corticotropin-releasing factor-binding protein.

The venom proteins were separated on 12% sodium dodecyl sulfate-polyacrylamide gel electrophoresis (SDS-PAGE) gels, with bands ranging from 10 to more than 250 kDa (apparent molecular mass, Figure 1), which we resolved into 922 proteins by LC-MS/MS. The peptide sequences of these proteins matched the VG and FAC unigene databases (Supplementary Table S2), among which 55 unigenes (Figure 2) were included in the UVG database (Supplementary Table S3). Some of these unigenes correspond to cellular proteins such as ribosomal proteins without predicted signal peptides (e.g., comp44503_c0, comp44417_c0, comp11049_c0, comp11512_c0, comp44398_c0, and comp44381_c0), which were likely contaminants from cell leakage during venom collection. Limiting the venom components in silico to the putative proteins with predicted signal peptides, or with venom homologs in other parasitoid species, totally resulted in 37 candidate venom proteins (Figure 2 and Table 3). Abundant venom proteins are usually more important [1]. The LC-MS/MS system (LTQ-VELOS; Thermo Finnigan, San Jose, CA, USA) and Sequest search algorithm we used could not provide us quantitative information exactly, so here we divided the venom components into three relative transcript levels according to RPKM values: (1) low abundance, RPKM < 100; (2) medium abundance, RPKM from 100 to 1000; and (3) high abundance, RPKM > 1000 (listed in Table 3). Nine components belong to the high-abundance group, including a carboxypeptidase and three esterases implicating the important roles of enzymes played in the venom cocktail. Thirteen venom components fall into low-abundance group, including a mesencephalic astrocyte-derived neurotrophic factor (MANF), two DnaJ homolog subfamily members and two endoplasmic reticulum proteins (ERp). These proteins may be mainly expressed in secretory cells and regulate venom protein folding and secretion. Whether these proteins are actual venom components or tissue contamination is still unclear. We validated the venom proteins by determining the tissue specificity of these venom gene transcription because these venom genes would be expressed exclusively, or nearly so, in the venom gland. Although some possible venom genes, such as FKBP14 (Figure 3E), IEP-2 (Figure 3H), CRT (Figure 3J) and CRF-BP (Figure 3K) were not specifically expressed in the venom gland, we did not exclude their possible roles as virulence factors when expressed in the venom gland, even at low concentration [36,44]. These data indicate specific venom tissue expression of most of the venom genes (Figure 3).

In the following sections we discuss the major groups of venom proteins.

#### 2.2.1. Hydrolases

##### Proteases

**Figure 4:**
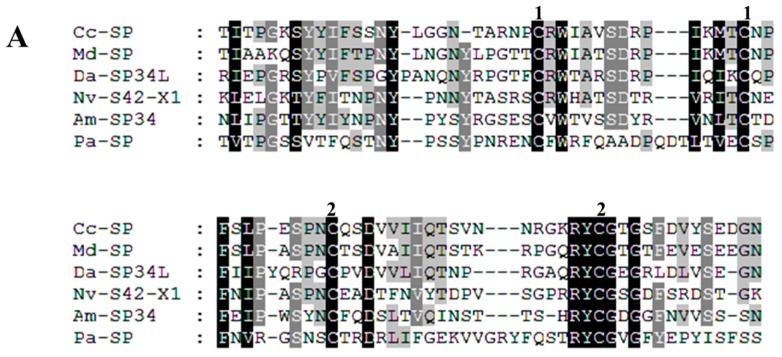

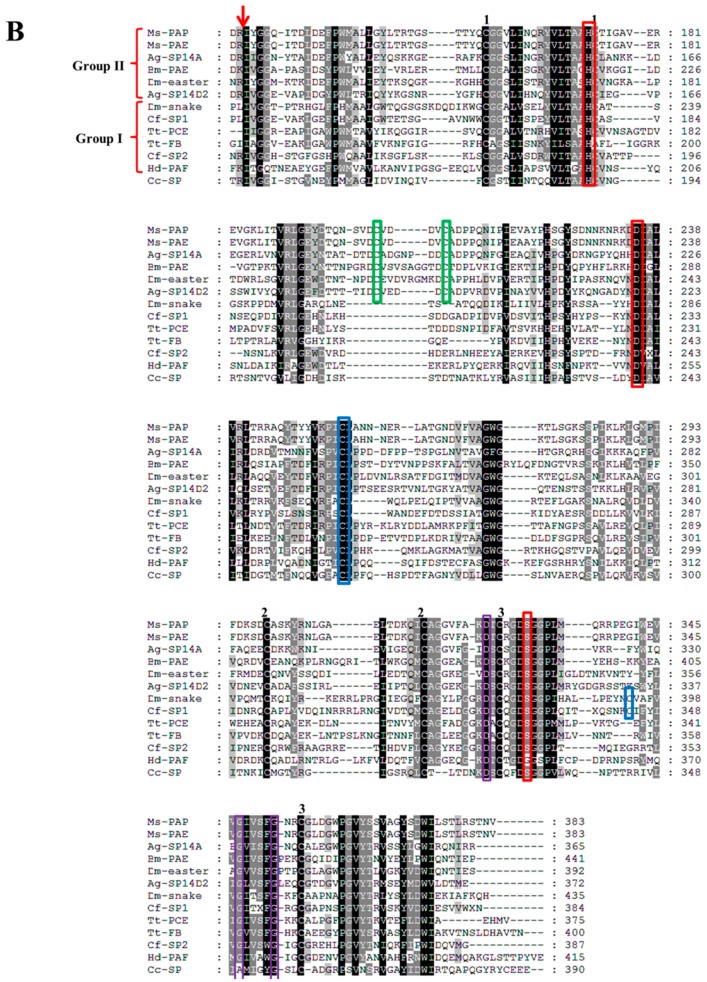
(**A**) Multiple sequence alignment of CUB domains of serine proteases. The paired numbers above the conserved Cys residues indicate two pairs of the disulfide linkages (**B**) Multiple alignment of the catalytic domains of arthropod serine proteases. The amino acids of the catalytic triad are in the red boxes and the determinants of the specificity pocket are in the purple boxes. The paired numbers above conserved Cys residues indicate the disulfide linkage in the catalytic domain. Cysteines involved in interdomain disulfide bonds are in the blue boxes. The two unique Cys residues in most group II proteases are shown in the green boxes. The amino acid prior to the proteolytic activation site (arrow) is included at the beginning of each sequence. The sequence analyses of catalytic domains were mainly based on [50]. Multiple alignment was performed using ClustalX2. Protein full names and sequence accession numbers are provided in Supplementary Table S4.

Serine protease (SP) Cc-Ven1 (comp32062_c0) shares high sequence similarity (BlastP, 50% identity, E-value = 2e^−130^) to SP homolog 42 isoform 1 precursor (GenBank: NP_001155078.1) identified from the venom of an ectoparasitoid wasp, *Nasonia vitripennis* (Hymenoptera: Pteromalidae). There were 15 members of the SP family screened from *Nasonia vitripennis* venom using bioinformatic and/or proteomic approaches. The SP family is the best represented of all *Nasonia vitripennis* venom constituents [46]. The functions of all *Nasonia vitripennis* venom SPs have not been thoroughly investigated. Bee venom SP kills insects via melanization and exhibits fibrin(ogen)olytic activity [47]. Serine protease homologs (SPH), formed by an amino acid substitution in the three catalytic triads (His-Asp-Ser), share similar amino acid sequences with active SPs, and function in several physiological processes of insects [9]. For instance, a venom SPH (Vn50) from *Cotesia rubecula* without proteolytic activity (because the conserved serine of the catalytic triad is glycine) significantly inhibited melanization of host hemolymph via suppressing prophenoloxidase activation. The deduced amino acid sequence of Cc-Ven1 is composed by two domains, including a CUB domain (cd00041, interval: 31–112, E-value: 1.93e^−04^) at N-terminus and a catalytic domain (cd00190, interval: 148–379, E-value: 2.20e^−53^) at C-terminus. The CUB domains are usually present in the proteins related to development [48]. This domain is commonly characterized by four conserved cysteine residues, forming two pairs of disulfide bonds (Figure 4A). Compared to the CUB-domain SPs (CUBs), the identifications and functions of Clip-domain SPs (CLIPs) are studied very well. The sequences of CUBs and CLIPs are similar with each other in their catalytic domains. According to this point, we did the multiple sequence alignment of Cc-Ven1 with other arthropod SPs, including CLIPs and CUBs, based on their catalytic domains [49]. In the catalytic domain, Cc-Ven1 also contains the three catalytic triads and three disulfide linkages, like the condition in the catalytic domains of other SPs. We found that Cc-Ven1 is more similar to Group I SPs. A difference between Group I and II SPs is that the cleavage sites in all Group ΙΙ members are followed with Lys or Arg, while the sites in most enzymes of Group Ι are followed by Leu or Ile. Additionally, the catalytic domains of Group II members contain a short insertion, including two specific Cys residues, present between the catalytic triads His and Asp (Figure 4B) [49]. Group Ι enzyme contains coagulation factor B and proclotting enzyme of *Tachypleus tridentatus* (Xiphosurida Limulidae), which indicates to us it may act in regulating host hemolymph clotting. However, Cc-Ven1 differs from non-venom SPs by the replacement of the determinant Gly by Ala in the specificity pocket, thus indicating that Cc-Ven1 has a distinct protease target specificity.

**Figure 5:**
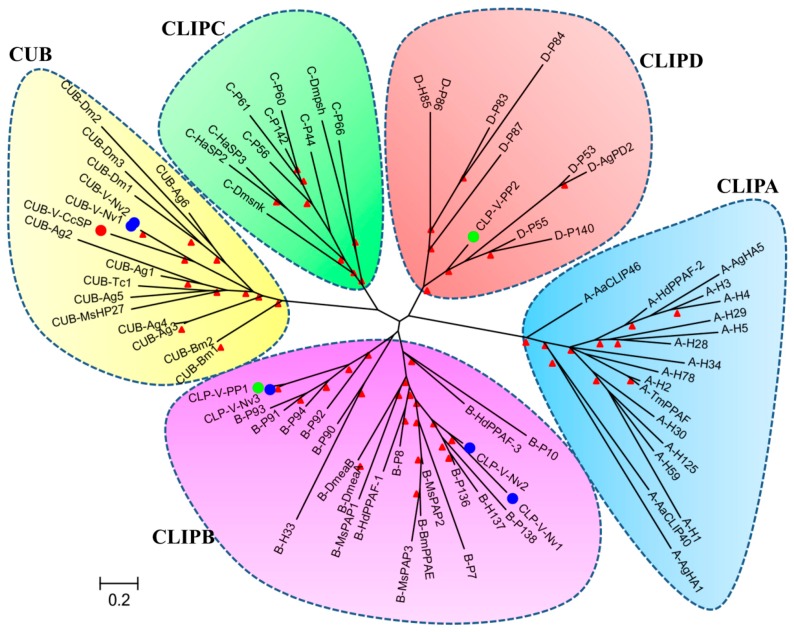
Phylogenetic tree built based on an alignment of the catalytic and protease-like domains of serine proteases and serine protease homologs. Maximum likelihood unrooted tree is based on deduced amino acid sequences of different catalytic and protease-like domains and constructed using PhylML with Smart Model Selection [58]. The best model is LG+G+I. Red arrowheads at nodes indicate bootstrap values higher than 50%. Circles with different colors represent the venom serine proteases from different parasitoid wasps (red: *Cotesia chilonis*; blue: *Nasonia vitripennis*; green: *Pteromalus puparum*). The full names of the proteins and accession numbers of the sequences are listed in Supplementary Table S4.

For the phylogenetic analysis, we chose a total of 57 of CLIPs and 13 of CUBs, respectively according to recently reported literature [51,52,53,54]. We also chose venom CLIPs and CUBs from two parasitoid wasps: *Nasonia vitripennis* and *Pteromalus puparum* [46,55]. According to [52,56], CLIPs are divided into four subfamilies. Subfamily A (CLIPA) is composed by SPHs solely, whereas subfamilies B (CLIPB), C (CLIPC), and D (CLIPD) comprise SPs, mainly. All of CUBs including the venom CUB, Cc-Ven1 (CUB-V-CcSP) are clustered into the same clade. Two venom CUBs from *Nasonia vitripennis* are clustered into the CUB clade. Three venom CLIPs from *Nasonia vitripennis* are clustered into CLIPB, while two venom CLIPs from *Pteromalus puparum* are clustered into CLIPB and CLIPD, respectively (Figure 5). Four CLIPs may represent lineages of SP derived from ancient evolutionary events since these subfamilies already exist in *Anopheles gambiae* (Diptera: Culicidae), *Drosophila melanogaster*, and *Helicoverpa armigera* (Lepidoptera: Noctuidae) [51,52,56]. Moreover, expansion of individual groups occurred several times to account for the gene clusters observed in *Tribolium castaneum* (Coleoptera: Tenebrionidae), *Anopheles* gambiae, and *Drosophila melanogaster* genomes [51,56,57]. The three venom CLIPs from *Nasonia vitripennis* might originate from SPs in CLIPB, whereas two venom CLIPs of *Pteromalus puparum* might originate from two different subfamilies (CLIPB and CLIPD) and were recruited to venom respectively. Cc-ven1 and two venom CUBs are clustered together in CUB clade, which indicates a similar function they probably achieve. Our phylogenetic analysis implies that the evolutionary processes of these venom SPs from parasitoid wasps may be strongly diverse. However, the essential functions of Cc-ven1 and other venom SPs are still unknown. We infer that venom SPs probably play different roles in the successful parasitization, including increasing the efficiency of venom, suppressing host immunity, and regulating the host development.

We identified three metalloproteinases belonging to the metzincin protease superfamily [59]. Cc-Ven2 (comp35977_c0) displays 29% identity (BlastP, E-value = 1e^−58^) to a disintegrin and metalloproteinase with thrombospondin motifs (ADAMTS) (GenBank: EGI57486.1) from the leaf-cutting ant, *Acromyrmex echinatior* (Hymenoptera: Formicidae). ADAMTS proteins are structurally organized into a proteinase and an ancillary domain and they are found in all metazoans, acting in the structure and function of the extracellular matrix [59]. ADAMTS occur in venoms from snakes, social stinging wasps and endoparasitoids, contributing to tissue damage [50,60]. Cc-Ven2 contains an ADAM cysteine-rich domain (ACR) (smart00608, E-value = 4.83e^−03^), which regulates metalloprotease activity [61], but lacks an ancillary domain responsible for substrate-binding preferences [59]. Truncated single-domain proteins acting as virulence factors are for other venoms. Lacking selectivity domains could make them broadly toxic [62,63].

**Figure 6:**
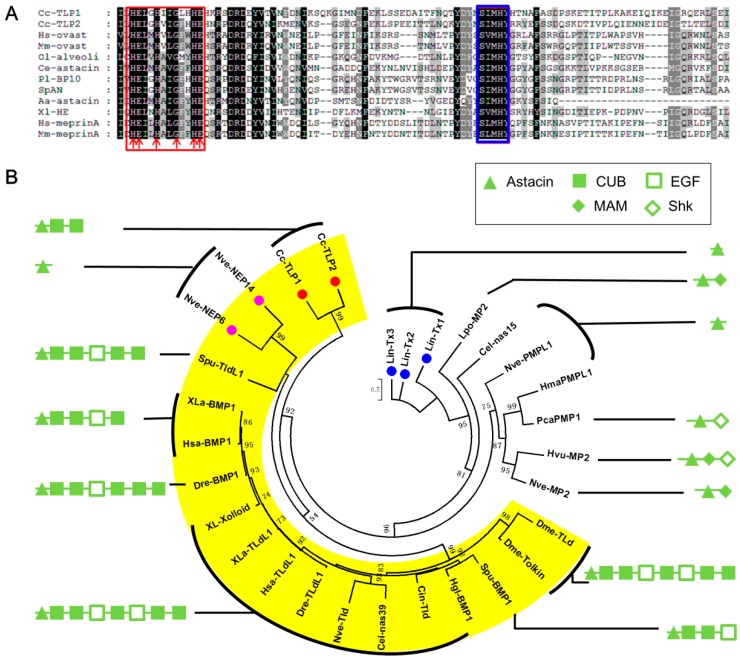
(**A**) Multiple alignment based on parts of catalytic domains of selected astacins. The alignment was performed using ClustalX2. Arrows in the red box point to the astacin signature sequence and blue box indicates the Met-turn; (**B**) Phylogeny of proteins containing Astacin domains. The maximum likelihood unrooted tree is based on deduced amino acid sequences of different Astacin domains and constructed using PhylML with Smart Model Selection [58]. The best model is LG+G+I. Bootstrap values higher than 50% are indicated at each corresponding node. Red circles indicate two astacin-like venom proteins from *Cotesia chilonis*. Purple and blue circles indicate astacin-like venom proteins identified from *Nematostella vectensis* and *Loxosceles intermedia* (Araneae: Sicariidae), respectively. BMP1/Tolloid subgroup of the Astacin family is shaded by yellow. The full names of the proteins and accession numbers of the sequences are listed in Supplementary Table S4.

Cc-Ven3 and Cc-Ven4 share 42% and 44% identity (BlastP, E-value = 1.00e^−94^ and 7.00e^−124^), respectively, with a Tolloid-like protein 1 (TLP 1) from *Apis florea* (GenBank: XP_003695260.2). They act in activation of growth factors, degradation of polypeptides, serve as venom toxins of brown spiders, digest molecules and spread other toxins through host bodies [64,65,66]. A typical Tolloid structure contains an N-terminal Astacin domain and C-terminal CUB domains [65], which determine specificity. Cc-Ven3 and Cc-Ven4 feature an Astacin domain (pfam01400) (Cc-Ven3: interval 45–245, E-value = 2.07e^−65^; Cc-Ven4: interval 45–238, E-value = 2.01e^−70^) (Figure 6A), and two CUB domains (pfam00431) (Cc-Ven3: interval 246–358, E-value = 3.11e^−15^ and interval 379–473, E-value = 1.42e^−28^; Cc-Ven4: interval 241–354, E-value = 2.25e^−22^ and interval 358–469, E-value = 1.13e^−28^). For phylogenetic analysis of proteins containing Astacin domains, we chose proteins according to the reference described by Moran et al. in 2013 [62]. Consistent with the previous results [62], two tolloid-like venom proteins of *Nematostella vectensis* (Actiniaria: Edwardsiidae), NEP-6 and NEP-14 without C-terminal CUB domains, which are different from typical Tolloid structure are clustered into the subgroup of Tolloid proteins, indicating evolved from a tolloid-like ancestor by loss of the CUB domains. By contrast, spider Astacin-like toxins are clustered out of the BMP1/Tolloid subgroup of the Astacin family, which indicates they are probably recruited into venom from other subgroups of Astacin family by losing other C-terminal domains, although the recruiting mechanism is not clear and need to be further studied in future. Here, the phylogenetic results show that Cc-Ven3 and Cc-Ven4 share a much closer evolutionary relationship with Cnidaria venom proteins than with spider venom proteins (Figure 6B). Although the reports of Astacin-like proteins as toxins are limited, the phylogenetic results imply that there may be a great diversity of evolutionary origins in arthropod venom proteins.

##### Peptidases

We identified two peptidases, CcVen5 and 6. Cc-Ven5 (comp11050_c0) shares 36% identity (BlastP, E-value = 5.00e^−71^) with a retinoid-inducible serine carboxypeptidase-like protein (GenBank: XP_001605442.1) from *Nasonia vitripennis*. CcVen5 contains a serine carboxypeptidase domain (pfam00450, E-value=6.29e^−27^). Cc-Ven6 (comp43465_c0; incomplete amino acid sequence) shares 41% identity (BlastP, E-value = 3.00e^−41^) to an aminopeptidase N-like protein (GenBank: EFN65598.1) from *Camponotus floridanus* (Hymenoptera: Formicidae) CcVen6 contains a peptidase_M1 domain (pfam01433, E-value = 8.50e^−09^). Peptidases are common components of parasitoid venoms [1]. Dipeptidyl peptidase IV (DDPIV) has been reported in the sting wasp, *Vespa basalis* (Hymenoptera: Vespidae) [67] and angiotensin-converting enzymes (ACEs) have been identified from the venoms of the parasitoid wasps *Chelonus inanitus* and *Nasonia vitripennis*, respectively [4].

##### Esterases

We identified five esterases. Cc-Ven7 (comp44319_c0) has 40% shared identity (BlastP, E-value = 1.00e^−43^) to 1-phosphatidylinositol phosphodiesterase-like protein from *Microplitis demolitor* (GenBank: XP_008561140.1). Cc-Ven8 (comp43453_c0) and Cc-Ven9 (comp43453_c1) share about 39% shared identity (BlastP, E-value = 4.00e^−52^; BlastP, E-value = 2.00e^−55^) to phosphatidylinositol-specific phospholipase C from the braconid *Microplitis demolitor* (GenBank: EZA44899.1). Cc-Ven7 and Cc-Ven9 contained a catalytic domain of *Bacillus cereus* (Bacillales: Bacillaceae) phosphatidylinositol-specific phospholipases C (PI-PLCc_BcPLC_like domain, cd08586, E-value = 5.60e^−20^ and 4.40e^−23^ respectively), and Cc-Ven8 has a catalytic domain of bacterial phosphatidylinositol-specific phospholipase C (PI-PLCc_bacteria_like domain, cd08557, E-value = 5.22e^−23^). Bacterial PI-PLCc acts in Ca^2+^-independent lipid phosphatidylinositol (PI) metabolism [68,69].

##### Glycosyl Hydrolase

Cc-Ven12 (comp35842_c0) has 57% sequence similarity (BlastP, 57% identity, E-value = 0) to a chitooligosaccharidolytic-β-*N*-acetylglucosaminidase (GenBank: XP_008213962.1) from *Nasonia vitripennis*. β-*N*-acetylhexosaminidases (*N*-acetylglucosaminidases, NAGs) catalyze the removal of β-1,4-linked *N*-acetyl-d-hexosamine residues from the ends of *N*-acetyl-β-d-hexosaminides including *N*-acetylglucosides and *N*-acetylgalactosides [70]. NAG is primarily responsible for the production of monomers from chitooligosaccharides for recycling [71].

NAGs have not been found in parasitoid venoms, but a 52-kDa chitinase was identified in the venom of the egg parasitoid *Chelonus* near *curvimaculatus* (Hymenoptera: Braconidae) containing all the conserved residues for chitinolytic activity. A role in dissociation and degradation of host tissue cells for endoparasitoid larval feeding has been proposed [72,73]. comp36742_c0 shows sequence similarity (BlastP, 88% identity, E-value = 0) to a chitinase domain-containing protein 1 from *Microplitis demolitor* (GenBank: XP 008553138.1) containing a Glyco_hydro_18 domain (pfam00704), corresponding to the glycosyl hydrolases family 18 (E-value = 3.17e^−21^).

##### Endonuclease

Cc-Ven13 (comp43476_c1) displays 60% identity (BlastP, E-value = 1e^−126^) with the enzymatic ortholog (endonuclease; reverse transcriptase) from the body louse *Pediculus humanus corporis* (Phthiraptera: Pediculidae) (GenBank: XP 002431503.1). Endonucleases have been reported in the venom of some snakes [74], *Physalia physalis* (Cnidaria: Siphonophora) [75], *Chrysaora quinquecirrha* (Cnidaria, Scyphozoa) [76], and *Nasonia vitripennis* [46].

#### 2.2.2. Isomerase

Cc-Ven14 (comp12229_c0) was originally identified as FK506-binding protein 2 (FKBP2) in the transcriptome. Its closest ortholog of six well characterized *Drosophila melanogaster* FKBPs is FKBP14 (GenBank: NP_476973.1), with 72% overall sequence identity (E-value = 6e^−105^) (Supplementary Table S5). For consistency with the literature, we rename Cc-Ven14 FKBP14 (Table 3). FKBPs belong to a family of highly-conserved proteins, immunophilins that bind to the immunosuppressive drugs FK506, rapamycin, and cyclosporin A. They often have peptidyl-prolyl cis-trans isomerase (PPIase) activity, which acts in a variety of functions, including protein folding and transcription [77,78]. FKBPs occur in insects, including *Spodoptera Frugiperda* (Lepidoptera: Noctuidae) [79], and *Manduca sexta* (Lepidoptera: Sphingidae) [80].

We totally identified two protein disulfide isomerases (PDIs) from the venom. Cc-Ven15 (comp32146_c0) has 76% identity (BlastP, E-value = 0) to PDI A6 (GenBank: XP_001602967.1) from *Nasonia vitripennis*. Cc-Ven16 (comp40525_c0) displays 49% identity (BlastP, E-value = 8e^−167^) with PDI (GenBank: XP_011138070.1) from *Harpegnathos saltator* (Formicidae: Ponerinae). PDIs are involved in the folding of the proteins containing disulphide bonds, via catalytic activities on disulfide bond formation and isomerization [81]. In some venomous organisms, PDIs have functions in the folding of toxin peptides [82,83]. For parasitoids, PDIs have been identified in the venom of *Aphidius ervi* [37] and *Psyttalia* species (Hymenoptera: Braconidae) [84], whose function remains to be elucidated.

#### 2.2.3. Extracellular Superoxide Dismutase 3

Cc-Ven17 (comp40765_c0) has 52% identity (BlastP, E-value = 2.00e^−56^) with the extracellular superoxide dismutase 3 (SOD3) from *Leptopilina boulardi* (GenBank: AET83769.1/AET83767.1). SODs catalyze dismutation of superoxide radicals generated via the electron transport chain or via dietary pro-oxidant compounds into molecular oxygen. The SOD3 gene is expressed specifically in venom tissue in *Leptopilina boulardi*, but not *Leptopilina heterotoma* [25,44].

#### 2.2.4. Serine Protease Inhibitors

Cc-Ven18 (comp23198_c0) displays 40% identity (BlastP, E-value = 3.00e^−22^) with the kazal-type protease inhibitor from *Camponotus japonicus* (GenBank: BAO48212.1) and Cc-Ven19 (comp23147_c0) displays 36% identity (BlastP, E-value = 3.00e^−71^) with a serine protease inhibitor from *Nasonia vitripennis* (GenBank: XP_008201843.1). Serine proteinase inhibitors have been described in several parasitoids, such as *Pimpla hypochondriaca* [85] and *Nasonia vitripennis* [46]. A *Leptopilina boulardi* venom serpin (LbSPNy) (Is_y_type) interferes with the PO activation in *Drosophila yakuba* (Diptera: Drosophilidae) host larva hemolymph [24].

#### 2.2.5. Other Venom Proteins

Alongside the enzymatic proteins mentioned above, we identified other components with high identities to the venom components previously characterized from other parasitoid wasps. Those are discussed in the following sections.

##### Immunoevasive Protein-2

Cc-Ven20 (comp39249_c0) and Cc-Ven21 (comp42303_c0) show 43% identity (BlastP, E-value = 1.00e^−62^) and 35% identity (BlastP, E-value = 5.00e^−13^), respectively, to immunoevasive protein-2 (IEP-2) (GenBank: BAB72015.1) from *Cotesia kariyai* (Hymenoptera: Braconidae). IEPs protect parasitoid eggs and *Cotesia kariyai* polydnavirus (CkPDV) from encapsulation by host hemocytes but not from hemocytes of larval cutworms, *Spodoptera litura* (Lepidoptera: Noctuidae), an incompatible *Cotesia kariyai* host [86]. The IEP protection for CkPDV depended on a thin surface layer covering virion particle [45].

##### CRT

Cc-Ven22 (comp11089_c0) has high similarity (BlastP, 98% identity, E-value = 0) to CRT from *Cotesia rubecula* (GenBank: AAN73309.1). CRT has been identified in several parasitoid venoms [34,36,46,87,88] and may act in many conserved roles contributing to the biology of parasites [88]. CRT from *Cotesia rubecula* (CrCRT) inhibited hemocyte-spreading behavior [33]. The coiled-coil domain helps CRT from *Pteromalus puparum* (PpCRT) enter *Pieris rapae* hemocytes, where it inhibits expression of host cellular immunity genes [89]. Siebert et al. used RNA interference directed to the venom CRT gene (*v-crc*) in *Nasonia vitripennis*, coupled with RNA-Seq in the envenomated insect host (vRNAi/eRNA-Seq) to investigate the *Nasonia vitripennis* v-crc, finding it inhibited melanization and reduced bleeding of the host during adult and larval parasitoid feeding [90].

##### Vascular Endothelial Growth Factor Receptor 2

Cc-Ven26 (comp23754_c0) displays 27% identify (BlastP, E-value = 7.00e^−13^) to vascular endothelial growth factor receptor 2 (VEGFR2) from the Indian jumping ant, *Harpegnathos saltator* (GenBank: EFN76191.1). VEGFR occurs in teratocytes of *Microplitis demolitor* [35].

##### Proteins Involved in the Folding and Export of Secreted Venom Proteins

Additionally, of the PDIs mentioned above, we also identified other proteins probably involved in the folding and export of secreted venom proteins. Cc-Ven27 (comp10689_c0), MANF is able to protect cells against the endoplasmic reticulum stress [91]. Cc-Ven28 (comp32038_c0) and Cc-Ven29 (comp45528_c0), annotated as DnaJ proteins, probably function as cofactors of heat shock proteins 70 kDa (Hsp70) and may modulate protein assembly, disassembly and translocation [92]. Cc-Ven30 (comp32086_c0), annotated as ERp29, is structurally similar to PDI and its function may be for protein folding and secretion [93], Cc-Ven31 (comp11316_c0), annotated as ERp44, probably functions as a multifunctional chaperone of the PDI family [94]. Cc-Ven32 (comp35875_c0) is annotated as a member of HSP70 family, and is one of the common chaperone of ubiquitous class. It is able to control the aspects of cellular proteostasis [95]. Hsp70 proteins were also identified in the venom of the parasitoids *Pteromalus puparum* [87] and two *Psyttalia* species [84], however, their functions are unknown. Actually, molecular chaperones were identified as venom proteins in some other parasitoid wasps [37,84]. For example, HSP70, calreticulin and PDI, were found in two *Psyttalia* species as venom components. It is speculated that they may play roles in the stabilization of other venom proteins, and/or their transport and targeting to the host cells [84]. Endoplasmins, another kind of molecular chaperones, have been identified from both *Aphidius ervi* and *Psyttalia* species venoms. It is reported that this protein is associated with the secretion of pancreatic lipases and their further internalization by intestinal cells in the rat [96]. It is speculated that it may help the secretion, stabilization, transport and host targeting of other venom proteins.

##### Other Unknown Proteins

Five *Cotesia chilonis* venom proteins were not similar to identified proteins, including Cc-Ven33: comp38220_c0; Cc-Ven34: comp44992_c0; Cc-Ven35: comp23465_c0; Cc-Ven36: comp39041_c2; Cc-Ven37: comp39175_c0. This is not surprising because many venom proteins have not been identified, as seen in *Nasonia vitripennis* [46], *Chelonus inanitus* [4], and *Microplitis demolitor* [35].

## 3. Materials and Methods

### 3.1. Insect Rearing

Laboratory cultures of *Chilo suppressalis* and *Cotesia chilonis* were reared under 28 ± 1 °C, 70%–80% relative humidity, and a 16 h light/8 h dark photoperiod as described [97]. After eclosion, adults were held in glass containers, fed ad libitum on 20% (*v*/*v*) honey solution.

### 3.2. Venom Gland Collection and Total RNA Isolation

Mated female wasps aged 1–3 days were anaesthetized at −70 °C for 5 min, swabbed with 75% ethanol (*v*/*v*), dried and then dissected in sterile Pringle’s phosphate-buffered saline (PBS) with 3 µL RNase inhibitor at 1 unit/µL (TOYOBO, Osaka, Japan) on an ice plate under a Leica MZ 16A stereomicroscope (Leica, Wetzlar, Germany). Venom glands and carcasses without venom apparatus were collected into TRIzol reagent (Invitrogen, Carlsbad, CA, USA). Total RNA was extracted using TRIzol reagent according to the manufacture’s protocol. We used agarose gel electrophoresis, Qubit Fluorometer (Thermo Scientific, Wilmington, DE, USA), Agilent 2100 (Agilent Technologies, Santa Clara, CA USA), and nanodrop 2000 (Thermo Scientific, Wilmington, DE, USA) to determine the quality and quantity of the total RNA samples, respectively.

### 3.3. Construction and Sequencing of cDNA Library

The construction and sequencing of cDNA library were done by Novogene Bioinformatics Institute (Beijing, China). Briefly, mRNA was purified from total RNA using poly-T oligo-attached magnetic beads. Under elevated temperature (94 °C), fragmentation was carried out using divalent cations in an Illumina proprietary fragmentation buffer. After first and second strand cDNA synthesis, the remaining overhangs were converted into blunt ends via exonuclease/polymerase and enzymes were removed. Illumina PE adapter oligonucleotides were ligated to prepare for hybridization following adenylation of 3′ ends of DNA fragments. The library fragments of preferentially 200 bp with ligated adaptors on both ends were selectively enriched using Illumina PCR Primer Cocktail in a 10 cycle PCR reaction. Products were purified (AMPure XP system) and quantified using the Agilent high sensitivity DNA assay on an Agilent Bioanalyzer 2100 system.

After cluster generation using TruSeq PE Cluster Kit v3-cBot-HS (Illumina, San Diego, CA, USA), the library preparations were sequenced on an Illumina Hiseq 2000 platform and 90 bp paired-end reads were generated.

### 3.4. Data Analysis

Raw reads in fastq format were processed through an in-house Perl scripts and then deposited into the NCBI Short Read Archive (SRA) database [98] by Novogene Bioinformatics (Beijing, China). We obtained the clean reads by removing the reads containing adapters, reads including ploy-N and low quality reads from the raw data. The remaining clean reads were assembled using Trinity v2012-10-05, as described for de novo transcriptome assembly without reference genome using the parameter min_kmer_cov set to two by default, and all other parameters set to default [43]. After assembling, the longest transcript from each transcript cluster was chosen as the unigene, according to the proposal recommended by Trinity [43]. All unigenes were annotated by blastx search with a cutoff of 1e^−5^ against the following databases: NCBI non-redundant protein sequences (Nr), NCBI non-redundant nucleotide sequences (Nt), Protein family (Pfam), Clusters of Orthologous Groups of proteins (KOG/COG), Swiss-Prot (a manually annotated and reviewed protein sequence database), KEGG Ortholog database (KO), and Gene Ontology (GO).

Gene expression levels were estimated with the software RSEM (rsem-1.2.0) for each sample. Clean data were mapped back onto the assembled transcriptome, and the readcount for each gene was calculated from the mapping results. Prior to differential gene expression analysis, for each sequenced library, the read counts were adjusted by edgeR (3.0.8) through one scaling normalized factor [99]. Differential expression analysis of two samples, including venom glands and carcasses, was performed using the R package DEGSeq v1.2.2 [100]. The *p*-values were adjusted to q-values to account for multiple testing [101]. The *q*-value < 0.005 and |log2(foldchange)| > 1 was set as the threshold for significantly-differential expression. GO enrichment analysis of the differentially-expressed genes was implemented by GOseq R based Wallenius non-central hyper-geometric distribution [102], which adjusts for gene length bias in DEGs. GO terms at the second level was used to perform GO annotation.

### 3.5. SDS-PAGE of Venom and Protein Identification

Mated female wasps, aged 1–3 days, were dissected in sterile Pringle’s PBS with 1 mM phenylmethanesulfonyl fluoride (PMSF) (Sigma, St. Louis, MO, USA) as described above. The venom reservoirs were isolated, washed three times, and transferred into 1.5 ml Eppendorf tubes. After centrifugation at 8000× *g* for 10 min at 4 °C, the supernatant was filtered with a 0.22 μm Millipore filter and stored at −70 °C until use. The concentration of venom protein was determined using the Modified Bradford Protein Assay Kit (Sangon Biotech, Shanghai, China).

Venom samples, 78 μg proteins, were separated on 12% SDS-PAGE gels and stained with Coomassie Brilliant Blue R-250. The SDS-PAGE and protein identification were performed by Shanghai Applied Protein Technology Co., Ltd. (Shanghai, China). The gel was cut into seven large sections, depending on the molecular masses of protein bands. Each gel slice was digested with trypsin, lyophilized and analysed on a LC-MS/MS system (LTQ-VELOS; Thermo Finnigan, San Jose, CA, USA). Samples were desalted on Zorbax 300 SB-C18 columns (Agilent Technologies, Wilmington, DE, USA) and then separated on a RP-C18 column (150 μm i.d., 150 mm length) (Column technology Inc., Fremont, CA, USA). Buffer A was water with 0.1% formic acid, buffer B was 84% acetonitrile with 0.1% formic acid. The buffer B gradient was: 0–50 min, from 4% to 50%; 50–54 min, from 50% to 100%; 54–60 min, 100%. The resulting MS/MS spectra were searched against the translated *Cotesia chilonis* transcriptome using the Sequest search algorithm [103]. Carbamidomethyl of cysteine and oxidation of methionine were set as fixed and variable modifications, respectively. Delta CN (≥0.1) and cross-correlation scores (Charge = 1, XCorr ≥ 1.9; Charge = 2, XCorr ≥ 2.2; Charge = 3, XCorr ≥ 3.75) were used to filter the peptide identification.

### 3.6. qPCR

The total RNA was isolated, separately, from wasp head, thorax, ovary, venom gland, and abdomen without ovary and venom gland as described above. cDNA was synthesized from 1 μg RNA using TransScript One-Step gDNA Removal and cDNA Synthesis SuperMix (Transgen, Beijing, China) for RT-PCR. Specific primers were designed with Primer 3 (http://bioinfo.ut.ee/primer3-0.4.0/) (Supplementary Table S6). qPCR was conducted using the CFX96™ Real-Time PCR Detection System (Bio-rad, Hercules, CA, USA). 28S rRNA, was used as a reference gene. qPCR was done in 25 μL reactions containing 12.5 μL SYBR^®^ *Premix Ex Taq™* II (Tli RNaseH Plus) (Takara, Otsu, Japan), 1 μL each primer (10 μM), 2 μL cDNA template, 8.5 μL sterile H_2_O, programmed at 95 °C for 30 s, followed by 40 cycles of 95 °C for 5 s and 60 °C for 30 s, followed by melting curve analysis.

The relative accumulations of mRNAs in each tissue was calculated using the comparative 2^−ΔΔCT^ method [104], following the guidelines of Bustin et al. [105]. We took the lowest mRNA abundance level as the calibrator and assessed the relative mRNA abundances by comparing the abundances of each target gene in other tissues to the lowest one. The results are presented as mean mRNA abundances of three independent biological replicates. mRNA abundances among tissues were compared using one-way analysis of variance (ANOVA) and Tukey’s test with statistical significance set at *p* < 0.05. All statistical analyses were performed using the Data Processing System (DPS) package (Version 9.5) [106].

### 3.7. Sequence Alignment and Phylogenetic Analysis

Prediction of signal peptide was performed by online software SignalP 4.1 [107]. Multiple sequence alignments of the amino acid sequences were performed with ClustalX2 [108] and edited with GeneDoc. For the phylogenetic analysis of CLIPs, we selected the sequences according to recent references [51,52,53], from different insect species including *Tribolium castaneum*, *Drosophila melanogaster*, *Anopheles gambiae*, *Bombyx mori* (Lepidoptera: Bombycidae), *Manduca sexta*, *Aedes aegypti* (Diptera: Culicidae), and *Helicoverpa armigera*. We selected CUBs from the same species described above. SPs of *Drosophila melanogaster*, *Anopheles gambiae*, *Bombyx mori* were obtained from NCBI RefSeq protein database, using Blastp with Cc-Ven1 as the query sequence. The sequences from database with the identities of E-value < e^−5^ were chosen. Additionally, we searched the Cc-Ven1 against the BeetleBase [109] to identify the SP of *Tribolium castaneum* (E-value < e^−5^). We used the library of Pfam HMM [110] to confirm the CUBs with a cutoff of 0.001. CUB of *Manduca sexta* we used was according to the reported literature [111]. Three venom CLIPs and one CUB (containing two isoforms) identified from *Nasonia vitripennis* [46], and two venom CLIPs from *Pteromalus puparum* [55] were also selected to construct the phylogenetic tree. For the phylogenetic analysis of proteins containing Astacin domains, the sequences we used were selected accordingly [62]. For the selected proteins used in analyses, not all proteins contained several domains (CUB, EGF, MAM, and Shk), but they all contained the common Astacin domain. The boundaries of catalytic domain (for SPs), proteinase-like domain (for SPHs), and Astacin domain were determined using Pfam. The multiple sequences containing the same domain were aligned using ClustalX2. For the catalytic and proteinase-like domains of SP and SPH, respectively, low quality alignment regions were removed by Gblocks Server [112]. PhyML with Smart Model Selection [58] was used to perform the phylogenetic analyses with 1000-fold bootstrap re-sampling. The full names of the proteins and accession numbers of the sequences used in this study are shown in Supplementary Table S4.

## 4. Conclusions

We offer several conclusions meant to provide a context for research into parasitoid venoms and their components. First, in their natural and in human-managed population dynamics, parasitoids provide a wide range of biological control services in agroecosystems. These services lead to reduced pest insect damage in cropping systems, reduced use of chemical insecticides, and they help slow the evolution of resistance in pest populations. It follows that research into all aspects of parasitoid biology, including biosystematics, ecology, physiology, biochemistry, and molecular biology contributes critically valuable new knowledge that can be applied to improve parasitoid efficacy. Recent research has led to new understandings and concepts in host-parasitoid relationships. Discovery of the immunosuppressive actions of parasitoid venoms and their active components guided the understanding that parasitoids have evolved specific mechanisms that operate to protect juvenile wasps from the dangerous host immunological reactions to overcome invaders. Looking forward, a large number of findings will drive research into the identities of specific venom components responsible for influencing various aspects of host biology.

Many parasitoid venom components, including proteins, have been reported [1]. Here, we report 37 putative venom proteins of *Cotesia chilonis* using the combination of transcriptomic and proteomic analyses. Our previous physiological research indicates that this parasitoid venom is able to partially inhibit host hemocyte-spreading behavior and encapsulation response to beads [42]. The results also imply that *Cotesia chilonis* uses a combination of passive and positive strategies to keep its offspring alive in its host larvae [42]. Consistent with the physiological results, we identified not only the proteins involved in active immune suppression, but also the proteins including IEP-2 and Crp32 in contribution to “passive” strategy as the previous descriptions. A passive strategy may include deposition of proteins to protect parasitoid eggs and young larvae. Three egg surface proteins have been identified, including IEP from *Cotesia kariyai*, Crp32 from *Cotesia rubecula* and an *O*-glycosylated protein called hemomucin from *Macrocentrus cingulum* (Hymenoptera: Braconidae), that protect the offspring from encapsulation during the early stages of growth [45,113,114]. Here, we report more evidence on the active and passive mechanisms used by *Cotesia chilonis* to avoid host immune responses.

Changing of selective binding sites of proteases probably alter the enzymes to be the toxins. The replacement of the determinant Gly by Ala in the specific pocket structure of SP (Cc-Ven1) and the loss of an ancillary domain responsible for substrate-binding specification of disintegrin and metalloproteinase (Cc-Ven2) could make them better candidates as virulence factors in the venom of *Cotesia chilonis*, as the condition in other noxious animal species [62]. However, for two Astacin-domain containing metalloproteinases (Cc-Ven3 and Cc-Ven4), the present CUB domains may improve their target specificity. This is probably why two TLPs from *Cotesia chilonis* venom possess relatively low similarity (only 49% of identities).

Without belaboring the point, the idea here is identifying potential biological functions of venom proteins almost immediately leads to testable hypotheses about specific mechanisms of host-parasitoid interactions. Various organisms, including insects, are sources of commercially- and/or biomedically useful proteins and chemistries. Workers in insect science grasp the economic significance of the soil bacterium, *Bacillus thuringiensis*, whose toxins and cognate genes have been highly developed into potent insect management technologies [115]. Commercially valued insect products include honey and pollination services from honey bees, *Apis mellifera* (Hymenoptera: Apidae), silk from the silkworm, *Bombyx mori*, shellac from the lac scale insect, *Laccifer lacca* (Homoptera: Coccidae), indelible iron gall ink from Oak-produced Aleppo galls triggered by the cynipid wasp, *Cynips gallae-tinctoriae* (Hymenoptera: Cynipidae), and the red cochineal dye from the scale *Dactylopius coccus* (Hemiptera: Dactylopiidae), formerly used as a fabric dye and now used in cosmetics [116]. The discovery of insect antimicrobial peptides in the early 1980s (>500 now known) has been developed into a research platform for development of medical antibiotics [117], a still on-going enterprise. Here, the point is that current and future research into parasitoid venoms will lead to discoveries of new chemistries, new proteins and new genes, some of which may become products useful in agriculture and many other areas.

Overall, we foresee research into parasitoid venom components will yield new understanding of parasitology, help generate new concepts and hypotheses and possibly lead to new valuable products.

## Figures and Tables

**Table 1:** Summary statistics of the analysis of the *Cotesia chilonis* venom gland and female adult carcass reads.

Transcripts/Unigenes	Raw Reads		Clean Reads		No. of Transcripts/Unigenes	N50	Meanlength
	Venom Gland	Female Adult Carcass	Venom Gland	Female Adult Carcass	-	-	-
Transcripts	67,097,344	63,686,328	65,515,114	62,221,866	115,957	2393	1254
Unigenes					71,617	1309	759

**Table 2:** Functional characterization of up regulated unigenes from *Cotesia chilonis* venom gland.

GO_Accession	Description	# Unigenes	Percentages *
GO:0003735	Structural constituent of ribosome	37	13.3
GO:0004553	Hydrolase activity, hydrolyzing *O*-glycosyl compounds	14	5.0
GO:0004620	Phospholipase activity	7	2.5
GO:0004629	Phospholipase C activity	6	2.2
GO:0005198	Structural molecule activity	42	15.1
GO:0008233	Peptidase activity	30	10.8
GO:0008237	Metallopeptidase activity	15	5.4
GO:0016798	Hydrolase activity, acting on glycosyl bonds	14	5.0
GO:0046982	Protein heterodimerization activity	3	1.1

* Percent values show the percent of upregulated VG unigenes (UVG).

**Table 3:** Proteins recorded from *Cotesia chilonis* venom.

Protein Name	RPKM/UniquePepCount	Putative Function	Sequence Length (Signal Peptide)	Blast Information (E-Value; Genbank No.; Species)	Gene ID	Accession No.
**Hydrolases**						
*Proteases*						
Cc-Ven1	106.52 (M)/2	Serine protease	390 (Y)	2e^−130^; NP_001155078.1; *Nasonia vitripennis*	comp32062_c0	KU663618
Cc-Ven2	47.99 (L)/9	Disintegrin and Metalloproteinase with thrombospondin motifs	509 (Y)	1e^−58^; EGI57486.1; *Acromyrmex echinatior*	comp35977_c0	KU663619
Cc-Ven3	212.13 (M)/11	Tolloid-like protein	478 (Y)	1.00e^−94^; XP_003695260.2; *Apis florea*	comp35892_c0	KU663620
Cc-Ven4	219.32 (M)/30	Tolloid-like protein	477 (Y)	7.00e^−124^; P_003695260.2; *Apis florea*	comp39295_c0	KU663621
*Peptidases*						
Cc-Ven5	1665.96 (H)/18	Retinoid-inducible serine carboxypeptidase-like	415 (Y)	5.00e^−71^; XP_001605442.1; *Nasonia vitripennis*	comp11050_c0	KU663622
Cc-Ven6	261.11 (M)/1	Aminopeptidase N-like	148 (NC)	3.00e^−41^; EFN65598.1; *Camponotus floridanus*	comp43465_c0	KU663623
*Esterases*						
Cc-Ven7	4876.97 (H)/9	1-phosphatidyl inositol phosphodiesterase precursor	230 (NC)	6.00e^−10^; XP 007809166.1; *Metarhizium acridum*	comp44319_c0	KU663624
Cc-Ven8	1010.49 (H)/30	Phosphatidylinositol-specific phospholipase	323 (Y)	4.00e^−52^; EZA44899.1; *Microplitis demolitor*	comp43453_c0	KU663625
Cc-Ven9	7010.08 (H)/29	Phosphatidylinositol-specific phospholipase	323 (Y)	2.00e^−55^; EZA44899.1; *Microplitis demolitor*	comp43453_c1	KU663626
Cc-Ven10	194.85 (M)/9	Phospholipase A1	295 (Y)	1.00e^−54^; EGI61859.1; *Acromyrmex echinatior*	comp11611_c0	KU663627
Cc-Ven11	427.00 (M)/14	Phospholipase A1	313 (Y)	6.00e^−89^; XP 011258702.1; *Camponotus floridanus*	comp35865_c0	KU663628
*Glycosyl hydrolase*						
Cc-Ven12	437.68 (M)/35	Chitooligosaccharidolytic β- *N*-acetylglucosaminidase	603 (Y)	0; XP_008213962.1; *Nasonia vitripennis*	comp35842_c0	KU663629
*Endonuclease*						
Cc-Ven13	24.39 (L)/1	Enzymatic polyprotein Endonuclease; Reverse transcriptase	1660 (NC)	1e^−126^; XP 002431503.1; *Pediculus humanus corporis*	comp43476_c1	KU663630
**Isomerase**						
Cc-Ven14	202.99 (M)/12	FK506-binding protein 14	239 (Y)	6e^−105^; NP_476973.1; *Drosophila melanogaster*	comp12229_c0	KU663631
Cc-Ven15	203.89 (M)/14	Protein disulfide-isomerase	2002 (Y)	0; XP_001602967.1; *Nasonia vitripennis*	comp32146_c0	-
Cc-Ven16	412.07 (M)/1	Protein disulfide-isomerase	3554 (Y)	8e^−167^; XP_011138070.1; *Harpegnathos saltator*	comp40525_c0	-
**Extracellular superoxide dismutase 3**						
Cc-Ven17	10.97 (L)/1	Extracellular superoxide dismutase 3	172 (Y)	2.00e^−56^; AET83769.1/AET83767.1; *Leptopilina boulardi*)	comp40765_c0	KU663632
**Serine protease inhibitors**						
Cc-Ven18	466.16 (M)/1	Kazal-type proteinase inhibitor	120 (Y)	3.00e^−22^; BAO48212.1; *Camponotus japonicus*	comp23198_c0	KU663633
Cc-Ven19	4970.74 (H)/16	Serine protease inhibitor (Serpin)	406 (Y)	3.00e^−71^; XP_008201843.1 *Nasonia vitripennis*	comp23147_c0	KU663634
**Other venom proteins (Non enzymatic and inhibitor activities)**						
Cc-Ven20	89.09 (L)/10	Immunoevasive protein-2	320 (NC)	1.00e^−62^; BAB72015.1 *Cotesia kariyai*.	comp39249_c0	KU663635
Cc-Ven21	103.23 (M)/7	Immunoevasive protein-2	180 (NC)	5.00e^−13^; BAB72015.1 *Cotesia kariyai*.	comp42303_c0	KU663636
Cc-Ven22	377.40 (M)/21	Calreticulin	403 (Y)	0; AAN73309.1; *Cotesia rubecula*	comp11089_c0	KU663637
Cc-Ven23	11187.79 (H)/34	Venom protein Ci-48a	384 (NC)	7.00e^−14^; CBM69271.1; *Chelonus inanitus*	comp39158_c0	KU663638
Cc-Ven24	1862.61 (H)/5	Icarapin-like precursor	227 (Y)	2.00e^−20^; NP 001012431.1; *Apis mellifera*	comp44327_c0	KU663639
Cc-Ven25	24.06 (L)/5	Corticotropin-releasing factor-binding protein	327 (Y)	3.00e^−95^; XP_003692566.1; *Apis florea*	comp25125_c0	KU663640
Cc-Ven26	36.45 (L)/8	Vascular endothelial growth factor receptor 2	231 (Y)	e^−13^; EFN76191.1; *Harpegnathos saltator*	comp23754_c0	KU663641
Cc-Ven27	36.81 (L)/11	Mesencephalic astrocyte-derived neurotrophic factor	842 (Y)	3.82e^−66^; BAM18078.1; *Papilio xuthus*	comp10689_c0	-
Cc-Ven28	59.91 (L)/1	DnaJ homolog subfamily C member 10-like	2650 (Y)	0; XP_001606269.2; *Nasonia vitripennis*	comp32038_c0	-
Cc-Ven29	64.12 (L)/3	DnaJ homolog subfamily B member 11-like	1665 (Y)	0; XP_624603.2; *Apis mellifera*	comp45528_c0	-
Cc-Ven30	67.36 (L)/6	Endoplasmic reticulum protein ERp29	987 (Y)	3.36e^−103^; EGI59372.1; *Acromyrmex echinatior*	comp32086_c0	-
Cc-Ven31	92.63 (L)/1	Endoplasmic reticulum resident protein 44	1350 (Y)	0; XP_624571.2; *Apis mellifera*	comp11316_c0	-
Cc-Ven32	101.69 (M)/52	Heat shock 70 kDa protein	2840 (Y)	0; EFN61604.1; *Camponotus floridanus*	comp35875_c0	-
Cc-Ven33	72.01 (L)/4	Unknown	357 (Y)	-	comp38220_c0	KU663642
Cc-Ven34	84.75 (L)/1	Unknown	132 (Y)	-	comp44992_c0	KU663643
Cc-Ven35	270.62 (M)/4	Unknown	516 (Y)	-	comp23465_c0	KU663644
Cc-Ven36	1465.76 (H)/20	Unknown	291 (Y)	-	comp39041_c2	KU663645
Cc-Ven37	1659.20 (H)/43	Unknown	1033 (Y)		comp39175_c0	KU663646

NC means that prediction of secretion could not be performed due to the incompleteness of the sequence and Y represents the deduced amino acid sequences with predicted signal peptides. L, M, and H represent low, medium and high abundance of unigene expression levels, respectively, according to the RPKM values.

## Supplementary Materials

The following are available online at www.mdpi.com/2072-6651/9/4/135/s1.
