# Supplementary material for: Protein Discovery: Combined Transcriptomic and Proteomic Analyses of Venom from the Endoparasitoid Cotesia chilonis (Hymenoptera: Braconidae)
Source: Toxins (Basel). 2017 Apr 12;9(4):135. doi: 10.3390/toxins9040135 (PMC5408209; doi:10.3390/toxins9040135)
Supplement: Supplementary file 1 [file toxins-09-00135-s001.zip › Supplementary table 6.pdf]

Supplementary table 6. Primers used for qPCR analysis of expression levels of putative venom genes in *C. chilonis*.

Primer name	Forward primer (5’-3’)	Reverse primer (5’-3’)
Serine protease	CGTCGATTTACTTGGCTGGG	TGGTGGGATTTTGCCACAAG
TLP	GACGATTCATTCAGCGGCTT	CGATGACATGTCCCAATTCGT
Phospholipase A1	TGGGTGCTCATATTCCTGCA	ACAAGGTCAAGTATCCGGCA
NAG	AACTCTTCGTCCATTCCCGT	TAAACAGGAGCGTCGGTGAT
FKBP14	TGTCACCCAATCTCTCGCTT	CGTACCGGTGTAATGCATGG
SOD3	CACTGGTACTCGTTTCTGCG	ATTGAAGTGTTCGCCAGTGC
Serpin	ACGCGAAAATCTTGTTGGCT	CGTCATTTTCATCGCCGGAT
IEP-2 (Cc-Ven20)	GTGTCATTGGTTCCAGCTGG	CAATGACTGCCTAAACCCGG
IEP-2 (Cc-Ven21)	CAACCCGGTTATGTTCAGCA	CATCGAAGCTCTTGACGCAT
Calreticulin	AAAGGACATGCACGGAGAGA	AGTCGGCTTCAAGTTCTCCA
CRFBP	GAACCTACAGTCTGCGGAGT	GTGGTCATTCTCTGGTGGGA
