# Supplementary material for: Protein Discovery: Combined Transcriptomic and Proteomic Analyses of Venom from the Endoparasitoid Cotesia chilonis (Hymenoptera: Braconidae)
Source: Toxins (Basel). 2017 Apr 12;9(4):135. doi: 10.3390/toxins9040135 (PMC5408209; doi:10.3390/toxins9040135)
Supplement: Supplementary file 1 [file toxins-09-00135-s001.zip › Supplementary table 4.docx]

**Supplementary table 4: The accession number of the sequences used in this study.**

**Figure 4**

**A. Multiple alignment of the CUB domains of serine proteases**

Md-SP: venom serine protease 34 from *Microplitis demolitor* (GeneBank accession no. XP_008558373.1)

Da-SP34L: according to reference [1]

Nv-S42-X1: serine protease homolog 42 isoform X1 from *Nasonia vitripennis* (GeneBank accession no. XP_008203180.1)

Am-SP34: venom serine protease 34 from *Apis mellifera* (GeneBank accession no. XP_392669.1)

Pa-SP: CUB-serine protease from *Panulirus argus* (GeneBank accession no. AAK48894.1)

**B. Multiple alignment of the catalytic domains of arthropod serine proteases.**

Ag-SP14A: serine protease 14A from *Anopheles gambiae* (GeneBank accession no. AAD38334.1);

Ag-SP14D2: serine protease 14D2 from *Anopheles gambiae* (GeneBank accession no. AAD38335.1);

Bm-PAE: prophenoloxidase activating enzyme from *Bombyx mori* (GeneBank accession no. NP_001036832.1);

Cf-SP1: trypsin-like serine protease from *Ctenocephalides felis* (GeneBank accession no. AAD21841.1);

Dm-easter: easter, isoform A from *Drosophila melanogaster* (GeneBank accession no. AAF55170.1);

Dm-snake: Serine protease snake from *Drosophila melanogaster* (GeneBank accession no. P05049.2)

Hd-PAF: prophenoloxidase activating factor from *Holotrichia diomphalia* (GeneBank accession no. CAC12665.1);

Ms-PAE: oxidase activating enzyme from *Manduca sexta* (GeneBank accession no. AAC64004.1);

Ms-PAP: prophenoloxidase-activating proteinase-1 from *Manduca sexta* (GeneBank accession no. AAX18636.1);

Tt-FB: coagulation factor B from *Tachypleus tridentatus* (GeneBank accession no. BAA03528.1);

Tt-PCE: proclotting enzyme from *Tachypleus tridentatus* (GeneBank accession no. AAA30094.1).

**Figure 5. The phylogenetic analysis of serine proteases in insects.**

*Tribolium castaneum* BeetleBase (http://www.bioinformatics.ksu.edu/BeetleBase/)

A-H1 GLEAN_00246

A-H125 GLEAN_11067

A-H2 GLEAN_00247

A-H28 GLEAN_01300

A-H29 GLEAN_01301

A-H3 GLEAN_00248

A-H30 GLEAN_01908

A-H34 GLEAN_02150

A-H4 GLEAN_00249

A-H5 GLEAN_00250

A-H59 GLEAN_04957

A-H78 GLEAN_07026

B-H137 GLEAN_13278

B-H33 GLEAN_02112

B-P10 GLEAN_00497

B-P136 GLEAN_13277

B-P138 GLEAN_13279

B-P7 GLEAN_00494

B-P8 GLEAN_00495

B-P90 GLEAN_09089

B-P91 GLEAN_09090

B-P92 GLEAN_09091

B-P93 GLEAN_09092

B-P94 GLEAN_09093

C-P142 GLEAN_13416

C-P44 GLEAN_04160

C-P56 GLEAN_04863

C-P60 GLEAN_05130

C-P61 GLEAN_05230

C-P66 GLEAN_05976

D-H85 GLEAN_08658

D-P140 GLEAN_13326

D-P53 GLEAN_04635

D-P55 GLEAN_04770

D-P83 GLEAN_08653

D-P84 GLEAN_08657

D-P86 GLEAN_08658

D-P87 GLEAN_08659

CUB-Tr1 GLEAN_09752

*Aedes aeypti* ImmunoDB http://cegg.unige.ch/Insecta/immunodb

A-AaCLIP40 CLIP40

A-AaCLIP46 CLIP60

*Anopheles gambiae* NCBI

A-AgHA1 >XP_320725.2

A-AgHA5 >XP_320729.4

D-AgPD2 >XP_317284.4

CUB-Ag3 >XP_001689277.1

CUB-Ag1 >XP_320615.4

CUB-Ag5 >XP_320619.4

CUB-Ag2 >XP_320620.4

CUB-Ag6 >XP_320621.4

CUB-Ag4 >XP_552329.3

*Drosophila melanogaster* NCBI

B-DmeaA >NP_001262597.1

B-DmeaB >NP_524362.2

C-Dmpsh >CAL85472.1

C-Dmsnk >NP_524338.2

CUB-Dm1 >NP_610370.1

CUB-Dm2 >NP_610366.1

CUB-Dm3 >NP_724665.1

*Holotrichia Diomphalia* NCBI

A-HdPPAF-2 >2B9L_A

B-HdPPAF-1 >2OLG_A

B-HdPPAF-3 >BAC15604.1

*Tenebrio molitor* NCBI

A-TmPPAF >CAC12696.1

*Bombyx mori* NCBI

B-BmPPAE >BAA76308.1

CUB-Bm1 >XP_004922529.1

CUB-Bm2 >NP_001155191.1

*Manduca sexta* NCBI

B-MsPAP1 >AAX18636.1

B-MsPAP2 >AAZ91696.1

B-MsPAP3 >AAX18637.1

CUB-MsHP27 HP27 (Reference)

*Helicoverpa armigera* Reference [2]

C-HaSP2 SP2

C-HaSP3 SP3

*Nasonia vitripennis* NCBI

CLP-V-Nv1 >XP_001600149.2

CLP-V-Nv2 >NP_001155077.1

CLP-V-Nv3 >NP_001155043.1

CUB-V-Nv1 >NP_001155078.1

CUB-V-Nv2 >NP_001155078.2

*Pteromalus puparum* Personal data

CLP-V-PP1 Personal data

CLP-V-PP2 Personal data

**Figure 6. A. Multiple alignment of part of catalytic domains of selected astacins. B. Phylogeny of proteins containing Astacin domains.**

Aa-astacin: zinc proteinase from *Astacus astacus* (GenBank accession no. CAB43519.1)

Ce-astacin: Zinc metalloproteinase nas-4 from *Caenorhabditis elegans* (GenBank accession no. NP_001254939.1)

Cel-nas15: nas15 from *Caenorhabditis elegans* (GenBank accession no. CAD99210)

Cel-nas39: nas39 *Caenorhabditis elegans* (GenBank accession no.Q20176)

Cin-Tld: tolloid from *Ciona intestinalis* (GenBank accession no.BAE06735)

Dme-Tld: tolloid-like *Drosophila melanogaster* (GenBank accession no.P25723)

Dme-TldR: tolloid-related-1 *Drosophila melanogaster (*GenBank accession no.AAA70057)

Dre-BMP1: bone morphogenic protein1 *Danio rerio* (GenBank accession no.AAI63535)

Dre-TldL1: tolloid-like *Danio rerio* (GenBank accession no.AAI63569)

Hgl-BMP1: bone morphogenic protein1 *Holothuria glaberrima* (GenBank accession no.AEP25603)

Hma-PMPL1: Podocoryne metalloproteinase-like1 *Hydra magnipapillata* (GenBank accession no.XP_002166518)

Hsa-BMP1: bone morphogenic protein1 *Homo sapiens* (GenBank accession no.AAA51833)

Hsa-TldL1: tolloid-like *Homo sapiens* (GenBank accession no.NP_036596)

Hs-MeprinA: meprin A subunit alpha precursor from *Homo sapiens* (GenBank accession no. NP005579)

Hs-ovast: oocyte astacin from *Homo sapiens* (GenBank accession no. CAD61265)

Hvu-MP2: bone morphogenic protein2 *Nematostella vectensis* (GenBank accession no.AAD33860)

Lin-Tx1: toxin *Loxosceles intermedia* (GenBank accession no.ABK20019)

Lin-Tx2: toxin *Loxosceles intermedia* (GenBank accession no.ACV52010)

Lin-Tx3: toxin *Loxosceles intermedia* (GenBank accession no.ACV52011)

Lpo-MP2: bone morphogenic protein2 *Limulus polyphemus (*GenBank accession no.CAQ16893)

Mm-MeprinA: meprin A subunit alpha from *Mus musculus* (GenBank accession no. NP032611)

Mm-ovast: oocyte astacin from *Mus musculus* (GenBank accession no. CAD61264)

Nve-MP2: bone morphogenic protein2 *Nematostella vectensis* (GenBank accession no.XP_001628402)

Nve-NEP14: Tolloid-related proteins14 *Nematostella vectensis* (GenBank accession no.XP_001623204)

Nve-NEP6: Tolloid-related proteins6 *Nematostella vectensis* (GenBank accession no.XP_001628963)

Nve-PMPL1: Podocoryne metalloproteinase-like1 *Nematostella vectensis* (GenBank accession no.XP_001629833)

Nve-Tld: Tolloid *Nematostella vectensis* (GenBank accession no.XP_001633846)

Ol-Alveolin: alveolin from *Oryzias latipes* (GenBank accession no. NP_001098139)

Pca-PMP1: Podocoryne metalloproteinase-like1 *Podocoryne carnea* (GenBank accession no.CAA06314)

Pl-BP10: blastula protease-10 from *Paracentrotus lividus* (GenBank accession no. CAA39673)

SPAN: SpAN protein from *Strongylocentrotus purpuratus* (GenBank accession no. AAA30072)

Spu-BMP1: bone morphogenic protein1 *Strongylocentrotus purpuratus* (GenBank accession no.P98069)

Spu-TldL1: tolloid-like Xenopus laevis *Strongylocentrotus purpuratus* (GenBank accession no.XP_782751)

XLa-BMP1: bone morphogenic protein1 *Xenopus laevis* (GenBank accession no.P98070)

XLa-TldL1: tolloid-like *Xenopus laevis* (GenBank accession no.NP_001083894)

XLa-Xolloid: Xolloid *Xenopus laevis* (GenBank accession no.AAI70044)

Xl-HE: hatching enzyme from *Xenopus laevis* (GenBank accession no. BAA14003.1)

**Supplementary data 4. Alignment of FKBP14 from venom of *Coteisa chiloins* with other FKBPs using blastp (**[**http://blast.ncbi.nlm.nih.gov/Blast.cgi**](http://blast.ncbi.nlm.nih.gov/Blast.cgi)**).**

Dm-CG14715: *Drosophila melanogaster* （GenBank accession no. NP_650101.1);

Dm-FK506-BP2: *Drosophila melanogaster* （Dm-FK506-BP2, GenBank accession no. NP_523792.2);

Dm-FKBP14: *Drosophila melanogaster*（GenBank accession no. NP_476973.1);

Gg-FKBP14: *Gallus gallus*（GenBank accession no. XP_418735.1);

Gg-FKBP1A: *Gallus gallus* (GenBank accession no. NP_989661.1);

Gg-FKBP7: *Gallus gallus*（GenBank accession no. XP_421981.4);

Hs- FKBP1A : *Homo sapiens* (GenBank accession no. NP_000792.1);

Hs-FKBP14: *Homo sapiens*（GenBank accession no.NP_060416.1);

Hs-FKBP2: *Homo sapiens*（GenBank accession no. AAH03384.1);

Hs-FKBP7: *Homo sapiens*（GenBank accession no. AAQ57208.1);

Mm- FKBP 1a: *Mus musculus* (GenBank accession no. NP_032045.1);

Mm- FKBP 2: *Mus musculus*（GenBank accession no. NP_032046.1);

Mm- FKBP 7: *Mus musculus*（GenBank accession no. NP_034352.1);

Mm-FKBP14: *Mus musculus*（GenBank accession no. NP_705801.1).

1. Levine, M.Z.; Harrison, P.J.H.; Walthall, W.W.; Tai, P.C.; Derby, C.D. A CUB-serine protease in the olfactory organ of the spiny lobster *Panulirus argus*. *J. Neurobiol.* **2001**, *49*, 277-302.

2. Xiong, G.-H.; Xing, L.-S.; Lin, Z.; Saha, T.T.; Wang, C.; Jiang, H.; Zou, Z. High throughput profiling of the cotton bollworm *Helicoverpa armigera* immunotranscriptome during the fungal and bacterial infections. *BMC Genomics* **2015**, *16*.
